# Lower Multiple Sclerosis Severity Score Is Associated with Higher Adherence to Mediterranean Diet in Subjects with Multiple Sclerosis from Northwestern Italy

**DOI:** 10.3390/nu16060880

**Published:** 2024-03-18

**Authors:** Matteo Bronzini, Alessandro Maglione, Rachele Rosso, Federica Masuzzo, Manuela Matta, Raffaella Meroni, Simona Rolla, Marinella Clerico

**Affiliations:** 1 Department of Clinical and Biological Sciences, University of Turin, 10043 Orbassano, Italy; matteo.bronzini@unito.it (M.B.); alessandro.maglione@unito.it (A.M.); rachele.rosso@unito.it (R.R.); raffaella.meroni@unito.it (R.M.); marinella.clerico@unito.it (M.C.); 2 Neurology Unit, San Luigi Gonzaga University Hospital, 10043 Orbassano, Italy; federicamasuzzo@gmail.com (F.M.); m.matta@sanluigi.piemonte.it (M.M.)

**Keywords:** multiple sclerosis, Mediterranean diet, MSSS, MEDAS, IMI, PyrMDS, food diary, fiber

## Abstract

The Mediterranean Diet (MD) is described in the literature as a beneficial dietary pattern for neurodegenerative diseases such as Multiple Sclerosis (MS). The objective of this study was to evaluate the dietary habits in people with MS (pwMS) and to test whether adherence to the MD could have an impact on the severity of the disease measured as the MS severity score (MSSS). Adherence to the MD was assessed in 31 PwMS using the Mediterranean Diet Adherence Screener (MEDAS), the Pyramid-based Mediterranean Diet Score (PyrMDS) index, and the Italian Mediterranean Index (IMI), and their eating habits were recorded in a food diary for a one-year follow-up. When data obtained from dietary analysis were compared to the MSSS, results showed that pwMS with lower MSSS adhere more to the MD than the other pwMS groups according to the MEDAS index. Furthermore, a high consumption of fiber in the MS mild severity class was observed. Further studies are needed to clarify which of the nutritional components of the MD may impact the course of MS and if the sensitization of pwMS to MD adherence can be a strategy for mitigating the disease.

## 1. Introduction

Multiple sclerosis (MS) is a chronic neuroinflammatory disease of the central nervous system characterized by neuronal degeneration associated with a high risk of physical disability [1,2]. MS is considered a complex disease since both genetic and environmental factors contribute to its onset. Within the latter, diet appears to play an important role in the disease course due to its impact on the intestinal microbiota, which in turn affects the immune system balance [3,4]. Diet was shown to influence the risk of diseases, including neurodegenerative ones [5]. Thus, the quality of the diet followed by people with MS (pwMS) can be relevant for the MS course [4].

Different types of diet can have diverse impacts on the pwMS’s health; a ketogenic diet, low-sodium intake diet, and fast mimicking diet were reported to reduce the peripheral lymphocyte count and to slightly decrease the Expanded Disability Status Scale (EDSS) score, to have a positive correlation with the exacerbation rates, and to promote a mild reduction in the EDSS scores in pwMS, respectively [4,6,7,8]. Among different diets, the Mediterranean Diet (MD) is largely described in the literature for its efficacy in preventing several chronic diseases, including neurodegenerative ones [9,10]. Adherence to the MD ameliorates the inflammatory status and the severity of symptoms such as cognitive dysfunction, fatigue, and attack rate in pwMS [11]. Also, higher adherence to the MD was associated with lower pwMS disability [12].

The MD is characterized by a high intake of vegetables, fruits, minimally refined cereals, beans, nuts, and seeds, a high intake of olive oil as the principal source of fat, a moderate intake of dairy products, and low red meat consumption [13]. Currently, one of the most used methods to estimate adherence to the MD includes the analysis of specific food questionnaires that people must complete by indicating their eating habits, from which specific adherence indices are then obtained.

In this study, we investigated the adherence to the MD, calculated using the Mediterranean Diet Adherence Screener (MEDAS) [14], Pyramid-based Mediterranean Diet Score (PyrMDS) [12], and Italian Mediterranean Index (IMI) [15] in pwMS from a cohort of northwestern Italy. Furthermore, dietary intakes were collected and analyzed using a 3-day food intake diary that pwMS filled in every 3 months for 1 year. The results of the three different scores were correlated with macronutrient intake, and adherence to the MD was associated with MS severity.

## 2. Materials and Methods

### 2.1. Subjects

Thirty-one pwMS were enrolled in the study at the SSD Patologie Neurologiche Specialistiche, Department of Clinical and Biological Sciences, University of Turin, Orbassano (TO) according to the following inclusion and exclusion criteria. Inclusion criteria: patients with a diagnosis of relapsing–remitting MS (RRMS) defined according to the most recent revision of the McDonald’s criteria [16]; age > 18 years; omnivore diet; ability to express informed consent. Exclusion criteria: any medical condition that does not allow the signing of informed consent; acute or chronic gastrointestinal diseases; eating disorders; diagnosis of other chronic, autoimmune, or neoplastic diseases; pregnancy or breastfeeding. This study obtained approval from the ethics committee of AOU San Luigi Gonzaga, Orbassano (TO), Italy. The patients/participants provided their written informed consent to participate in this study.

### 2.2. Mediterranean Diet Questionnaires

At recruitment, the following questionnaires were administered with a nutritionist interview:

- Fifteen item Pyramid-based Mediterranean Diet Score (PyrMDS) [17], which consists of 15 items and adopts the strategy of introducing different and continuous weights for both under- and over-consumption, but in a different way for each food item in order to quantify how they fit the 2011 version of the MD pyramid [14,18]. To estimate the adherence to the MD based on the PyrMDS, we used an open web-based tool [19].
- Fourteen item Mediterranean Diet Adherence Screener (MEDAS) [20], which assesses typical consumption of healthful foods (e.g., olive oil, vegetables) and avoidance of unhealthy foods (e.g., red meat, butter). Patients receive a score of 1 or 0 for each item which is summed to derive a MEDAS score.
- Eleven item Italian Mediterranean Index [15] that summarizes the intakes of 6 typical Mediterranean foods, 4 non-Mediterranean foods, and alcohol. Components and standard portions for optimal scoring of each index were previously described [15].

### 2.3. Food Diaries

Dietary intake was assessed using a 3-day food intake diary. Participants were instructed by a nutritionist to not modify usual dietary habits, to record after every meal, to write ingredients of recipes, to report names of products written on labels for industrial foods, and to quantify foods using spoons and cups or estimate using their hand. They had to record everything they ate and drank, including supplements and medications. Participants were instructed to write a 3-day food intake diary, every 3 months for 1 year. Analysis of dietary intake was done for 6 non-consecutive days in the year covering 4 weekdays and 2 weekend days. The 4 weekdays have been chosen randomly among the days from Monday to Friday, during all the seasons of the year (1 day in autumn, 1 in winter, 1 in summer, and 1 in spring), to consider all the possible variations of the seasonal intakes and without excluding holidays periods (summer holidays, Christmas, etc.). The 2 weekend days have been chosen randomly in a way that 1 day belongs to the autumn–winter period and 1 to the spring–summer period without considering annual festivities (Easter, Christmas). In this way, a more realistic average annual intake can be represented.

Names of foods and quantities were input in an Excel file, and specific outcomes were output with the Food Composition Database for Epidemiological Studies in Italy [21]. These data were used to determine whether participants met for some nutrients the Suggested Dietary Target (SDT), Population Reference Intake (PRI), and Reference Intake (RI) of Reference Intake Levels of Nutrients and Energy for the Italian Population [22].

### 2.4. MS Clinical Scores

The Expanded Disability Status Scale (EDSS) is the most widely used clinical scale to quantify disability in MS. It is based on a standard neurological examination, during which the functionality of neurological systems (i.e., muscle weakness or difficulty moving limbs, ataxia, loss of balance, coordination or tremor, problems with speech, swallowing and nystagmus, numbness or loss of sensations, bowel and bladder function, visual impairment, memory impairment) are rated using an ordinal clinical rating scale ranging from 0 (normal neurologic examination) to 10 (death due to MS) in half-point increments [23]. The EDSS of each subject was assessed at the time of enrollment in the study.

The Multiple Sclerosis Severity Score (MSSS) was developed by Roxburgh et al. and can be used to compare disease progression using single assessment data over the course of the disease [24]. The Multiple Sclerosis Severity Score (MSSS) for each subject was obtained using the global MSSS as a reference table, taking into account the subject’s EDSS and disease duration, thus indicating the clinical severity of MS at the time of observation.

### 2.5. Statistical Analysis

In the descriptive statistics, quantitative variables were expressed by mean and standard deviation (SD) or median and interquartile range (IQR), while qualitative variables were expressed by percentages and frequencies. Student’s *t*-test or one-way ANOVA was used to test differences between groups using GraphPad software version 8.2.1 (18 August 2019). Logistic regression analysis was performed to study the association between MS severity, computed with MSSS, and level of adherence to the MD computed by MEDAS. The *p*-values were calculated for a multivariate model adjusted for age, sex, BMI, and physical activity level (sedentary, partially active, active). Differences with *p* ≤ 0.05 were regarded as statistically significant. Logistic regression analysis has been performed in the R environment with R version 3.6.3 (29 February 2020).

## 3. Results

### 3.1. Dietary Pattern of the Population

Participants had a mean EDSS of 1.45 (±1.2) and a mean MS disease duration of 2.7 years (±4.3). Clinical and lifestyle data of pwMS participating in this study are shown in Table 1. The mean values describe a population with a mean BMI of 24 (±4.4). When examining daily dietary intake, females had lower energy intake than males with statistical significance (*p* = 0.017). The mean values of protein intake were higher than the recommended intake for the Italian population (LARN) of 0.9 g/Kg (PRI, [22]), three pwMS had lower protein intake (two 0.8 g/kg and one 0.7 g/kg), males had lower protein intake than females with statistical significance (*p* = 0.027). The mean intake of simple sugar (SS F 16.4; M 14.7) and fatty acids (FA F 35.3; M 36.8) were upper borderlines for both sexes considering the limits of 15% for SS (SDT, [22]) and 35% for FA (RI, [22]). For saturated fatty acids (SFA), the mean levels (females 11.5; males 12.5) were higher than the SDT of 10% [22]. Total dietary fiber intake was below the recommendation (>25 gr die and 12.6–16.7 g/1000 kcal, RI, [22]) for both sexes, but the levels of males were lower than females with statistical significance (*p* = 0.011 for gr fiber/1000 Kcal). Considering the MD alignments, MEDAS, PyrMDS, and IMI means were higher in females compared to males, but the differences were not statistically significant (MEDAS *p* = 0.153; PyrMDS *p* = 0.326; IMI *p* = 0.304).

Participants were divided into quartiles based on the percentage of adherence to the MD calculated using MEDAS, PyrMDS, and IMI; 93.6% for MEDAS, 96.7% for PyrMDS, and 90% for IMI of this population study were in Q2 and Q3. The distribution of participants is described in Table 2.

**Figure 1:**
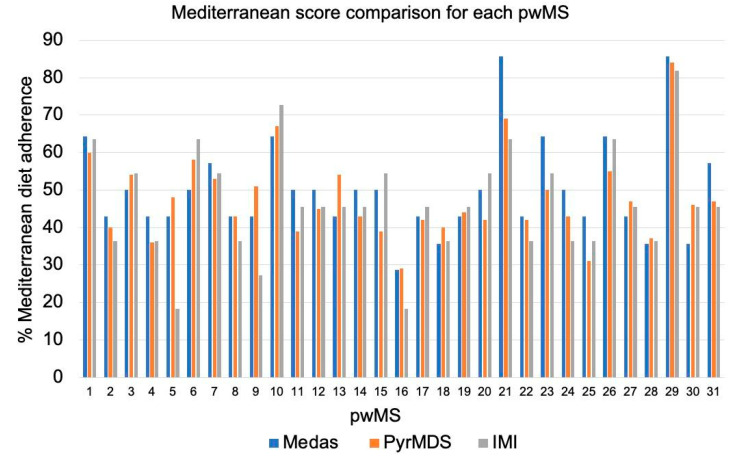
Bar plots represent the scores of each of the 31 pwMS for MEDAS (blue), PyrMDS (orange), and IMI (grey) scores.

The 61.3% of pwMS were in the same quartile for every one of the three scores, and the others differed by just one quartile of one score. Figure 1 describes the differences between the three scores for each participant. When the delta of adherence between the higher and the lower score for each patient was computed, four pwMS (number 5, 9, 15, and 21) were in the 90 percentile, thus having a delta higher than 15.5%. This higher variability is dependent on the different questions in each questionnaire (Supplementary Table S1).

**Figure 2:**
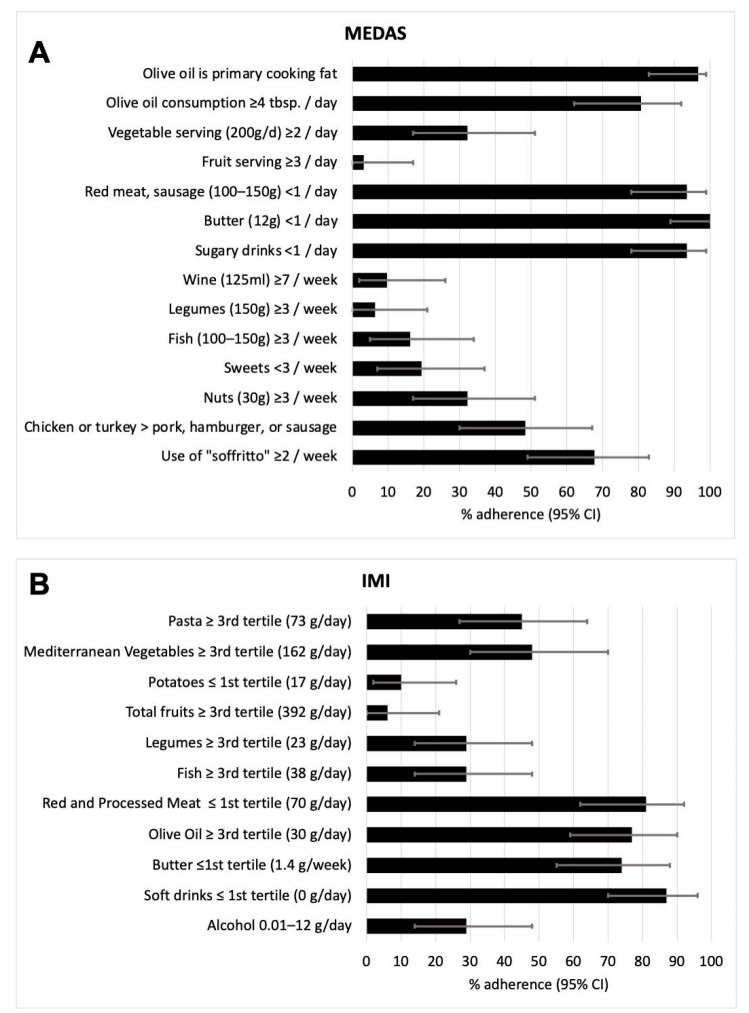
Bar plots represent % of adherence (95% CI) of patients to each of the questions included in the MEDAS questionnaire (**A**) and IMI questionnaire (**B**).

To understand which specific food contributes to determining the degree of adherence to MD, we tested the MEDAS and IMI single item. On average, pwMS of our cohort showed low adherence regarding the consumption of vegetables (32% MEDAS score), fruits (3% MEDAS score, 6% IMI score), legumes (6% MEDAS score, 29% IMI score), fish (16% MEDAS score, 29% IMI score), potatoes (10% IMI), nuts (32% MEDAS score), sweets (19% MEDAS score), and wine/alcohol (10% MEDAS score, 29% IMI). Conversely, we observed high adherence regarding the consumption of olive oil (97% MEDAS score, 77% IMI score), butter (100% MEDAS score, 74% IMI score), sugary drinks/soft drinks (94% MEDAS score, 87% IMI score), and red and processed meat (81% IMI score) (Figure 2) [22].

### 3.2. Adherence to MD Positively Correlates with Fiber and Protein Intake and Is Negatively Correlated with Saturated Fats Intake

**Figure 3:**
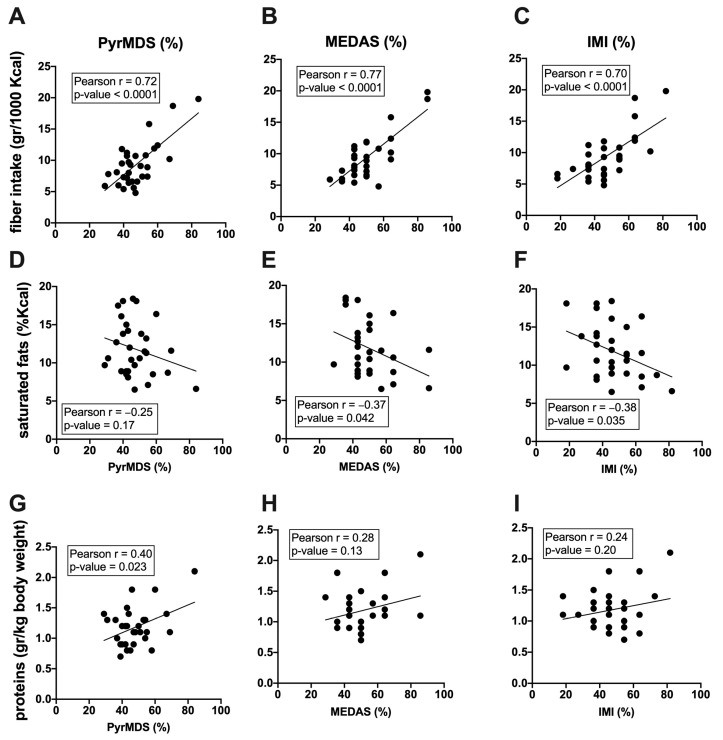
Correlations between macronutrients and PyrMDS, MEDAS, and IMI are represented. Pearson correlation between fiber intake (gr/1000 Kcal) and PyrMDS (**A**), MEDAS (**B**), and IMI (**C**), between saturated fats (gr/1000 Kcal) and PyrMDS (**D**), MEDAS (**E**), and IMI (**F**) and between proteins (gr/kg of body weight) and PyrMDS (**G**), MEDAS (**H**), and IMI (**I**) are reported. For each correlation plot, the best linear fitting, r, and *p*-values are reported.

To assess whether the results from the IMI, MEDAS, and PyrMDS, which are based on the MD food groups, match with the real food intake of our cohort, we correlated MD adherence scores with mean daily dietary intake of macronutrients obtained from food diaries. We found a significant positive correlation between fiber intake and PyrMDS (Figure 3A, r = 0.72, *p* < 0.0001), MEDAS (Figure 3B, r = 0.77, *p* < 0.0001), and IMI (Figure 3C, r = 0.70, *p* < 0.0001) scores. A significant positive correlation was also found between protein intake and PyrMDS (Figure 3G, r = 0.40, *p* = 0.023), although to a lesser extent. Moreover, saturated fat intake was found to have a significant negative correlation with MEDAS (Figure 3E, r = −0.37, *p* = 0.042) and IMI (Figure 3F, r = −0.38, *p* = 0.035). No correlation was found between MD adherence scores and every other macronutrient tested, including carbohydrates, sugars, and total fats (Supplementary Figure S1).

### 3.3. Adherence to MD Is Associated to a Milder Disease Severity

**Figure 4:**
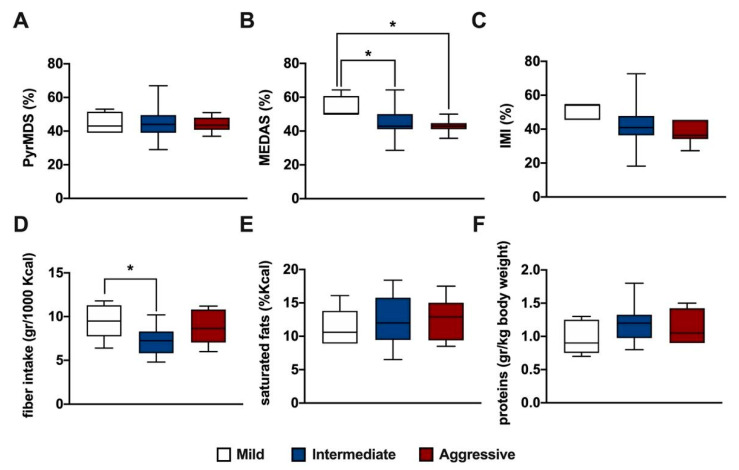
Box plots represent the distribution of PyrMDS (**A**), MEDAS (**B**), IMI (**C**) scores, fiber (**D**), saturated fats (**E**), and proteins intake (**F**) in pwMS divided into three classes of disease severity according to their MSSS: mild (MSSS 0–1.7), intermediate (MSSS 1.7–5), and aggressive (MSSS > 5). Statistical significance was assessed by one-way ANOVA with Bonferroni correction for multiple comparisons. * indicates a *p*-value ≤ 0.05.

We next investigated MD adherence and macronutrient intake with disease severity. PwMS were divided into three classes of disease severity according to their MSSS: mild (MSSS 0–1.7), intermediate (MSSS 1.7–5), and aggressive (MSSS > 5). A difference in MEDAS score was found to be statistically significant between mild, intermediate (Figure 4A, ANOVA, *p* < 0.05), and aggressive (Figure 4A, ANOVA, *p* < 0.05) MS courses. Moreover, a significant difference in fiber intake was found between mild and intermediate (Figure 4D, ANOVA, *p* < 0.05) MS courses.

Logistic regression analysis was performed, adjusting for confounding factors, including age, sex, BMI, and physical activity (Table 3). This analysis confirmed that higher adherence to the MD, expressed by the MEDAS score, was associated with MSSS (*p* = 0.02).

## 4. Discussion

The present study aimed to investigate the relationship between adherence to the MD, dietary intake, and MS disease. The key finding indicates that higher adherence to the MD and higher intake of fiber is associated with lower MSSS.

Overall, the dietary patterns of our cohort were similar to the average per capita supply of the Italian population and to the amounts of food group available for consumption corrected for waste in Italy [25]. This data was confirmed in a previous investigation of dietary intake between PwMS and health control [26,27,28]. Regarding MD adherence, PwMS from the Northwest of Italy showed low intakes of some healthy Mediterranean foods such as vegetables, fruits, legumes, nuts, and fish and a moderate consumption of wine. Low intakes of unhealthy foods such as red and processed meat, sugary/soft drinks, and butter were reported. By contrast, almost all participants used olive oil as their main cooking fat and dressing.

Assessing adherence to MD is not a matter of course, as various questionnaires have been developed over the years. In this regard, these three serving-based questionnaires have been critically evaluated, but a standardized evaluation method has not yet been identified [14]. Portion-based questionnaires show differences between them, both in the individual items considered and in the optimal consumption thresholds. Furthermore, some questionnaires adopt a single threshold and do not consider the proximity of the frequency to the ideal target or over-consumption (e.g., MEDAS, IMI). On the contrary, others set an optimal consumption range and adopt a gradual variation of the values attributed in proportion to the degree of under- or over-consumption of different foods (e.g., PyrMDS) [14]. Therefore, different MD adherence questionnaires can produce different estimates of MD adherence. Notably, MEDAS has already been used to correlate MD adherence to clinical MS outcomes [12] and correlates with three-day food diaries in Italy, Greece, and Portugal [29].

Comparing PyrMDS, IMI, and MEDAS, the three questionnaires used in this work, we observed that some foods (or groups of food) are present in all indexes (olive oil, fruits, legumes, fish, red and processed meat, alcohol/wine), some in two of three (butter, vegetables, potatoes, nuts, soft drinks, sweets), and some in just one of the three (sofrito, Mediterranean vegetables, whole grains, pasta, poultry, dairy, eggs). When dietary intakes from the 3-day food diary were analyzed in terms of macronutrients, we found that higher MD adherence scores were significantly associated with higher intake of fiber and proteins and less intake of saturated fats. This result was not surprising as the MD is primarily a plant-based dietary pattern characterized by a high abundance of fruit, vegetables, legumes, and whole grains, which are rich in fiber, and a low abundance of processed meat that is a source of saturated fats [13]. So, fiber intake may be a useful, but not unambiguous, value in determining whether or not adherence to the MD is maintained.

PyrMDS, but not IMI and MEDAS, better captured the intake of proteins, an indicator of nutritional status; this may be due to its gradual variation of the values based on portions of foods with respect to IMI and MEDAS, that are single threshold questionnaires. As for saturated fats, on the other hand, IMI and MEDAS showed a correlation, while PyrMDS did not correlate. This could be related to the fact that PyrMDS considers the consumption of dairy products, which are a source of saturated fats, increasing the MD adherence score (i.e., the optimal score is reached with two portions per day).

The regression analysis found an association between the MEDAS score, but not the PyrMDS and IMI, and the MSSS. PwMS with mild disease severity had a higher MEDAS score compared to other severity classes (intermediate and aggressive). Moreover, the intake of fiber was significantly different between mild and intermediate classes of disease severity, suggesting a role of this macronutrient in affecting disease severity. Interestingly, the aggressive class included pwMS with high disease activity at the onset, and they all received high-efficacy therapy. Even if they consumed a similar amount of fiber compared to the mild group, their MEDAS was lower. This could be related to their low consumption of olive oil and sofrito, as assessed by MEDAS (questions 2 and 14, respectively), reinforcing the evidence that the MD can be beneficial for pwMS considering all the food groups.

The main rationale for adhering to the MD in MS includes the presence of nutrients and their relative food sources that can modulate neuroinflammation and influence gut microbiota composition. The MD dietary pattern is characterized by the use of olive oil and an abundance of fruit, legumes, and vegetables, which are naturally rich in fiber and antioxidants, such as vitamins and polyphenols, but also fish and nuts, rich in omega-3 fatty acids. Olive oil is rich in polyphenols [30], and its use in the MD is widespread. Polyphenols have antioxidant, anti-inflammatory, and neuroprotective effects. They have the ability to cross the blood–brain barrier and exert direct effects in the central nervous system or indirect effects via interaction with the gut microbiota [31]. Interestingly, soffritto or sofrito is a common Mediterranean culinary recipe according to which onion and garlic are fried in olive oil. The use of sofrito when cooking is considered beneficial as the presence of olive oil improves the bioaccessibility of the polyphenols, and the onion protects other food from oxidation, enriching the MD serving with these useful components [32]. The use of sofrito as a preparation technique is investigated only by MEDAS but not by IMI and PyrMDS. Additionally, MEDAS has two other peculiarities: the presence of three questions referring to olive oil (in quantities, predominant use, and sofrito) and the highest cut-off regarding consumption of fish (100–150 gr at least three times a week). This means that MEDAS, compared with IMI and PyrMDS, increases its value more in people with regular unrestricted consumption of olive oil and high fish intake. The inclusion in a diet of fish and plant-derived omega-3 fatty acids has been shown to be potentially beneficial in counteracting neuroinflammation in MS [33].

Our findings of an association between fiber consumption and milder MS severity may be consistent as dietary fiber fermentation is the major source of SCFAs [e.g., acetate, propionate, and butyrate]. SCFAs have been associated with immune-modulatory effects, such as neutrophil chemotaxis, cytokine expression, and Treg differentiation, thus regulating inflammation. Moreover, several SCFA-producing bacteria have been shown to be reduced in the MS gut microbiome, raising the hypothesis of an association between gut dysbiosis, fiber consumption, and MS disease [4].

The findings of this study agree with those of both international and Italian previously published studies. In the US, MD adherence in a cohort of pwMS was investigated using MEDAS, while disease progression and disability were assessed with MS Functional Composite (MSFC), which evaluates cognition, upper extremity coordination and gait speed, and patient-reported outcomes (PROs), including disability outcomes, depression, and anxiety. As a result of this study, higher adherence to the MD predicted better MS outcomes. An association between olive oil consumption, but also to greater consumption of nuts and wine, and lower consumption of pastries and sugary beverages was found. Depression was worse among patients who use butter, drink sugary beverages, and consume less fish [12]. Also, a Greek study showed that enhanced MD adherence, assessed by MedDietScore, a 29-item self-administered questionnaire including life, dietary habits, and consumption frequency of Mediterranean foods, was independently associated with lower prevalence of disease severity, lower depression, anxiety, less cognitive impairment, and disability [34].

In Italy, MD adherence was investigated using the MedDietScore in a cohort of Southern Italian pwMS. The authors found an inverse correlation between EDSS and MSSS but not with the total number of relapses [35]. Another recent Italian study evaluated the adherence to the MD in pwMS using the Medi-Lite score and found that higher adherence to the MD was associated with a higher probability of having a less aggressive MS, according to our findings. However, in this study, none of the single food groups was individually associated with MS severity [36].

A limitation of this study is that the subjects belong to a specific geographical area, thus not permitting the generalization of the results, and the small sample size. However, our cohort is composed of pwMS at the onset or with low disease duration, highlighting the role of the MD in regulating MS severity at the very beginning of the disease without confounding factors, including disability, comorbidities, and cognitive impairment that could influence dietary habits.

The use of both MD adherence questionnaires in combination with the 3-day food intake diary collected every 3 months for 1 year is a strength of this work as the collection of dietary data over a long period of time is able to better represent the eating habits of participants compared to one-time food frequency questionnaires [37]. Responses to one-time questionnaires can lead to untruthful reports (underestimated or overestimated) due to recall bias or social desirability. Nevertheless, in this study, the analysis of 3-day diaries was consistent with MD adherence questionnaires.

A limitation of 3-day food diaries, as previously noted in healthy populations [38,39], is the substantial effort required to document information accurately by participants, and we cannot exclude an underestimation of intakes, although a comparison of the intakes every 3 months did not show differences in terms of mean energy intake over 3 days.

## 5. Conclusions

Higher adherence to the MD, measured by MEDAS, was associated with disease severity. This association has not been found using PyrMDS and IMI, highlighting that different MD adherence questionnaires can produce different estimates and results. Further studies are needed to clarify which of the nutritional components of the MD, such as fiber or other foods (i.e., olive oil, fish) or their synergic activity may have a key impact on the course of MS. Sensitizing pwMS to MD adherence can be a future holistic strategy in mitigating disease course and comorbidities.

## Figures and Tables

**Table 1:** Patient characteristics.

	Female	Male	*p*-Value ^3^
**n ^1^**	20 (64.5)	11 (35.5)	
**Age ^2^**	40.3 (12.6)	36.2 (8.5)	0.388
**Body Mass Index ^2^**	23.2 (4.7)	25.8 (3.4)	0.112
**Smoking Status: current smokers ^1^**	5 (20)	6 (54.5)	0.107
**Physical Activity**			
Sedentary ^1^	10 (50)	4 (36.4)	0.482
Partially active ^1^	4 (20)	5 (45.4)	0.144
Active ^1^	6 (30)	2 (18)	0.488
**Current MS therapy**			
Dimethyl fumarate ^1^	10 (32.2)	7 (22.6)	
Cladribine ^1^	10 (32.2)	4 (12.9)	
**EDSS ^2^**	1.6 (1.3)	1.1 (0.9)	0.335
**MSSS ^2^**	3.5 (2.4)	2.86 (2.34)	0.460
**Disease duration ^2^**	3.6 (5.2)	1.5 (1.5)	0.272
**Macronutrients**			
Energy Kcal ^2^	1804.3 (364)	2120.7 (263.9)	0.017
Protein g/kg ^2^	1.3 (0.3)	1 (0.2)	0.027
Carbohydrate % En ^2^	47 (5.3)	46.6 (6.9)	0.856
Simple Sugar % En ^2^	16.4 (4.2)	14.7 (4.9)	0.327
Fat % En ^2^	35.3 (4.4)	36.8 (6.8)	0.477
Saturated fat % En ^2^	11.5 (3.3)	12.4 (4.2)	0.519
Fiber g ^2^	18.3 (6.1)	15.6 (4.5)	0.200
Fiber g/1000 Kcal ^2^	10.5 (3.8)	7.4 (2.1)	0.011
**Adherence to Mediterranean diet**			
MEDAS/14 ^2^	7.35 (2.1)	6.4 (0.9)	0.153
PyrMDS/15 ^2^	7.38 (2)	6.74 (1)	0.326
IMI/11 ^2^	5.35 (1.7)	4.73 (1.3)	0.304

^1^ % of the total; ^2^ mean (standard deviation); ^3^ *p*-value of Student’s *t*-test; En = Energy Kcal.

**Table 2:** Patient distribution according to their adherence to MD.

**MEDAS**	**Q1**	**Q2**	**Q3**	**Q4**
% of participants	0	48.4 (15)	45.2 (14)	6.4 (2)
Average range value		40.5	55.1	85.7
Median value range		42.9	50.0	85.7
Interquartile Range		(35.7–42.9)	(50–64.3)	85.7
**PyrMDS**	**Q1**	**Q2**	**Q3**	**Q4**
% of participants	0	64.5 (20)	32.2 (10)	3.2 (1)
Average range value		41.15	57.1	84
Median value range		42	54.5	84
Interquartile Range		(29–47)	(50–67)	
**IMI**	**Q1**	**Q2**	**Q3**	**Q4**
% of participants	6.4 (2)	58.1 (18)	32.2 (10)	3.2 (1)
Average range value	18.2	40.4	60.0	81.8
Median value range	18.2	41.0	59.1	81.8
Interquartile Range	18.2	(27.3–45.5)	(54.5–63.6)	

**Table 3:** Regression analysis.

Association between MSSS and Adherence to MD			
Variable	Beta Coefficients	Standard Error	*p*-Value
**Age (Years)**	0.07111	0.04149	0.1037
**Sex** Female Male	Ref 0.09695	1.03061	0.9261
**BMI**	−0.13093	0.11207	0.2579
**Physical activity** Sedentary Partially active Active	Ref −1.46971 −1.26458	1.06682 1.13506	0.1852 0.2799
**MEDAS**	−0.13303	0.05605	0.0290 *

* indicates a *p*-value ≤ 0.05 for a multivariate model adjusted for age, sex, BMI, and physical activity level.

## Data Availability

Inquiries can be directed to the corresponding author.

## Supplementary Materials

The following supporting information can be downloaded at: https://www.mdpi.com/article/10.3390/nu16060880/s1, Table S1: Foods questioned in MEDAS, IMI, and PyrMDS indexes; Figure S1: Correlations between macronutrients and pyrMDS, MEDAS, and IMI.
