# Supplementary material for: Lower Multiple Sclerosis Severity Score Is Associated with Higher Adherence to Mediterranean Diet in Subjects with Multiple Sclerosis from Northwestern Italy
Source: Nutrients. 2024 Mar 18;16(6):880. doi: 10.3390/nu16060880 (PMC10976244; doi:10.3390/nu16060880)
Supplement: Supplementary file 1 [file nutrients-16-00880-s001.zip › nutrients-2908554-supplementary.pdf]

Table S1. Foods questioned in MEDAS, IMI and PyrMDS indexes

Foods investigated	Medas Score	IMI	PyrMDS
**Olive oil**	+	+	+
**Butter**	+	+	
**Soffritto**	+		
**Vegetables**	+		+
**Mediterranean Vegetables**		+	
**Fruits**	+	+	+
**Whole grains**			+
**Pasta**		+	
**Potatoes**		+	+
**Nuts**	+		+
**Legumes**	+	+	+
**Fish**	+	+	+
**Red meat and processed meat**	+	+	+
**Poultry**			+
**Dairy**			+
**Eggs**			+
**Soft drinks**	+	+	
**Sweets**	+		+
**Alcohol/wine**	+	+	+

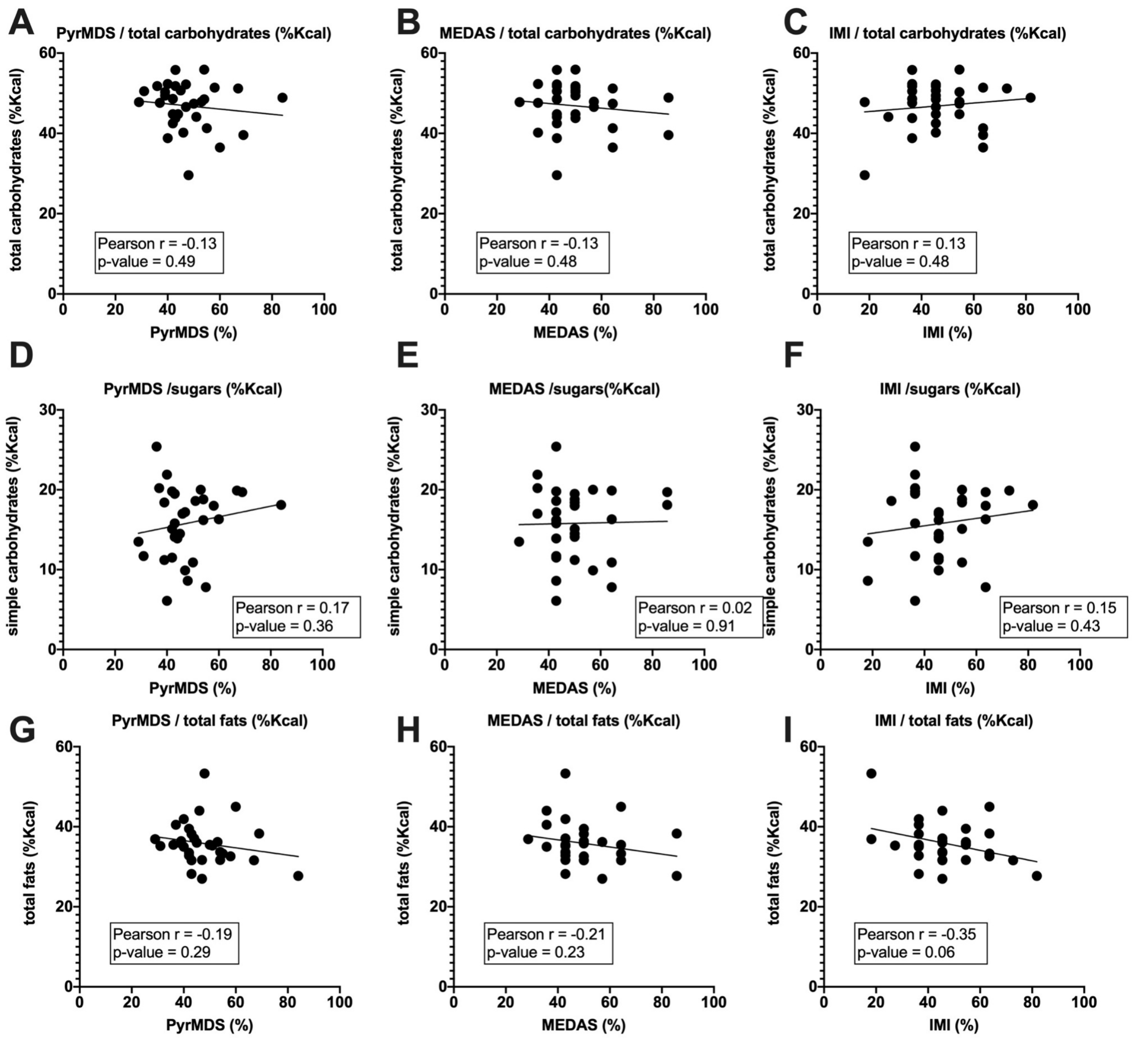

Figure S1. Correlations between macronutrients and pyrMDS, MEDAS and IMI. Pearson correlation between total carbohydrates (% Kcal) and pyrMDS (A), MEDAS (B) and IMI (C), between sugars (% Kcal) and pyrMDS (D), MEDAS (E) and IMI (F) and between total fats (% Kcal) and pyrMDS (G), MEDAS (H) and IMI (I) are reported. For each correlation plot the best linear-fitting, r and p-values are reported.
